# Therapeutic evaluation of electrochemical therapy combined with local injection of pingyangmycin for the treatment of venous malformations in the tongue

**DOI:** 10.3389/fneur.2024.1425395

**Published:** 2024-08-21

**Authors:** Weili Yuan, Xukai Wang

**Affiliations:** ^1^Department of Stomatology, The Fourth Affiliated Hospital of China Medical University, Shenyang, Liaoning, China; ^2^Department of Oral and Maxillofacial Surgery, School of Stomatology, China Medical University, Shenyang, Liaoning, China

**Keywords:** electrochemical therapy, pingyangmycin, venous malformation, tongue, sclerotherapy

## Abstract

**Background:**

Venous malformations are congenital developmental abnormalities that consist of enlarged dysplastic blood vessels. The tongue is a common site of venous malformations in the head and neck region. The aim of the present study was to evaluate the therapeutic effect of using electrochemical therapy (ECT) combined with local injection of pingyangmycin (PYM) for venous malformations in the tongue.

**Methods:**

60 patients (20 male and 40 female; age range, 8 to 68 yr) with venous malformations in the tongue were treated with a combination of ECT and PYM injection or with local injection of PYM alone in the department of oral and maxillofacial surgery of the stomatological hospital of China Medical University from January 2013 through June 2019. Among them, 30 patients (group A) were treated with ECT combined with PYM injection for tongue venous malformations and 30 patients (group B) were treated with local injection of PYM alone for tongue venous malformations. The size of the lesions in the two groups varied from 3.5 × 3 × 3 to 8 × 6 × 5 cm. There was no statistical difference in the volume of the lesions between group A and group B (*p* > 0.05). There was no statistical difference in the age between the two groups (*p* > 0.05). A repeated treatment of ECT combined with PYM injection or PYM injection alone was administered for venous malformations of tongue in the group A and group B. According to the size of the lesions, the amount of ECT was 5–10 C/cm^2^. The injection dose of PYM was 3 ~ 5 mL and the injection concentration of PYM was 1.6 mg/mL for adults and 1.0 mg/mL for children. Glucocorticoids were given to prevent postoperative swelling. The therapeutic interval was 3 months for ECT combined with PYM injection and 2 to 4 weeks for PYM injection alone. Hemisphere measurements were used to measure the size of the lesions. 4-scale score and feedback from the patients were used to evaluate the clinical efficacy.

**Results:**

During the follow-up period from 6 months to 3 years, 60 patients achieved different degree of improvement, with a total effective rate of 85%. 30 patients in the group A received ECT combined with local injection of PYM, with an effective rate of 97%. 30 patients in the group B received local injection of PYM alone, with an effective rate of 73%. The effectiveness of ECT combined with PYM injection in the group A was significantly higher than that of PYM injection alone in the group B (*p* < 0.05). Postoperative symptoms such as local pain, swelling and fever often occurred in the patients, and the symptoms generally disappeared after 5 to 7 days. No mucosa necrosis or nerve damage was found. Postoperative subjective sensation and function of the tongue were normal.

**Conclusion:**

Electrochemical therapy combined with local injection of pingyangmycin was a reliable, safe and minimally invasive method for the treatment of venous malformation in the tongue. The treatment modality has fewer complications and is worth of further promotion in clinic.

## Introduction

Vascular malformation is a kind of vascular abnormality caused by the abnormal morphology of vascular tissue, which is composed of blood vessels with abnormal arrangement of endothelial cells (1). The abnormal blood vessels involved could be classified into capillary malformations, venous malformations, arteriovenous malformations, lymphatic malformations, and mixed malformations (2). Tongue is a common site of venous malformation in the head and neck. Typical symptoms of venous malformations of the tongue are bleeding, pain and swelling as well as impairments of speaking, swallowing or even breathing (3). In the past, the treatment of tongue venous malformations included laser therapy, conventional surgery, sclerotherapy, interventional embolization and other methods, but the indications of the above methods were limited and the effect of clinical treatment was not ideal. In recent years, our team used electrochemical therapy combined with local injection of pingyangmycin or local injection of pingyangmycin alone in the treatment of tongue venous malformations in 60 patients in the department of oral and maxillofacial surgery of the stomatological hospital of China Medical University. We conducted a retrospective case–control study of the efficacy of the two therapeutic methods, as reported below.

## Methods

### Study design and participants

60 patients (20 male and 40 female; age range, 8 to 68 yr) with venous malformations in the tongue were treated with a combination of ECT and PYM injection or with local injection of PYM alone in the department of oral and maxillofacial surgery of the stomatological hospital of China Medical University from January 2013 through June 2019. A random sequence was generated using a computer program to assign the 60 patients in a 1:1 ratio to the two groups (group A and group B) of 30 patients each. The patients in the group A were treated with ECT combined with local injection of PYM for tongue venous malformation and the patients in the group B were treated with local injection of PYM alone for tongue venous malformation. There was no statistical difference in the age between the two groups (*p* > 0.05). The size of the lesions in the two groups varied from 3.5 × 3 × 3 to 8 × 6 × 5 cm, as measured by Doppler ultrasonography. There was no statistical difference in the volume of the lesions between group A and group B (*p* > 0.05). Venous malformations occurred in both the body and the lateral margin of tongue in 23 cases (38%). Venous malformations occurred solely in the body of tongue in 3 cases (5%). Venous malformations occurred solely in the lateral margin of tongue were 7 cases (12%). Venous malformations occurred solely in the abdomen of tongue were 12 cases (20%). Venous malformations occurred in both the abdomen and the lateral margin of tongue in 4 cases (7%). Multiple venous malformation of tongue occurred in 11 cases (18%). The demographics and relevant treatment of the patients in the two groups were shown in Table 1. All the lesions were ≥ 3 cm in diameter. There were 15 patients with high-return flow venous malformations in the group A and 8 patients with high-return flow venous malformations in the group B. The clinical manifestations of tongue venous malformations were as follows: (1) The lesions protruded from the surface of the tongue; (2) The surface mucosa of the lesions was blue-purple; (3) The lesions were soft and could be compressed, and the high-return flow venous malformations can be restored to their original state after being compressed for 5 s. We can confirm the diagnosis by asking the history, palpating the lesion site, taking enhanced CT, and examining magnetic resonance. None of the patients received any treatment before the surgery. The research plan was reviewed and approved by the medical ethics committee of the Stomatological Hospital of China Medical University. The treatment plan was approved by the patients and their families, and informed consent was signed prior to the treatment.

**Table 1:** Summary of the demographics and relevant treatment of the patients.

Demographics	*n* (%)
Gender	
Male	20 (33)
Female	40 (67)
Age (years)	
Mean	39.8
Range	8–68
Follow-up duration (months)	
Median	15
Range	6–36
Age at treatment initiation (years)	
<18	6 (10)
≥18	54 (90)
Maximum diameter of the lesion (cm)	
<5	25 (42)
≥5	35 (58)
Location	
The body of tongue	3 (5)
The lateral margin of tongue	7 (12)
Both the body and the lateral margin of tongue	23 (38)
The abdomen of tongue	12 (20)
Both the abdomen and the lateral margin of tongue	4 (7)
Multiple venous malformation of tongue	11 (18)
Previous treatment	0 (0)

### Inclusion and exclusion criteria

In order to ensure the patients’ safety, the inclusion criteria should be strictly followed as below: (I) the patient had not received any other drug treatment before the treatment; (II) according to the patient’s history and clinical characteristics, combined with color Doppler ultrasound, enhanced CT or MRI, the patient was diagnosed as having the tongue venous malformation; (III) the results of chest anteroposterior and lateral radiographs, electrocardiogram, blood routine test, coagulation test, and liver and kidney function tests were normal; (IV) the patients with complete clinical data had good compliance and were able to complete various examinations; (V) the patients and their families signed informed consent before the treatment.

The exclusion criteria were any of the following: (I) according to the classification of vascular diseases proposed by Waner and Suen (2), hemangioma and other vascular malformations were present. (II) the patients with acute inflammation or mucositis of the tongue; (III) the patients with platelet crisis or coagulation dysfunction; (IV) the patients with severe liver and kidney dysfunction; (V) a history of drug allergy to pingyangmycin or bleomycin.

### Therapeutic instruments and drugs

The therapeutic instrument used was VECHT-2 electrochemical hemangioma therapeutic apparatus (Number AOL-07004, Beijing Weili Heng Technology Development Co., LTD.), platinum electrode needle (diameter 0.7 cm, unipolar length 15 cm, bipolar length 5 cm); The sclerosing agent used was pingyangmycin hydrochloride injection (8 mg/dose, Jilin Aodong Pharmaceutical Group Yanji Co., LTD.).

### Treatment methods

#### Preparation of pingyangmycin injection solution

For tongue venous malformation, the usual injection concentration for adults was 1.6 mg/mL. The preparation of pingyangmycin injection solution (1.6 mg/mL) was as follows: 8 mg pingyangmycin dissolved in 5 mL mixed solution of lidocaine and normal saline (lidocaine mixed with 0.9% NaCl solution at a ratio of 1: 1); The injection concentration for children was 1.0 mg/mL. The preparation of pingyangmycin injection solution (1.0 mg/mL) was as follows: 8 mg pingyangmycin was dissolved in 8 mL mixed solution of lidocaine and normal saline (lidocaine mixed with 0.9% NaCl solution at a ratio of 1: 1), and the single injection dose was 3–5 mL. We have stated that there was no vasopressor drugs, such as epinephrine in the lidocaine since the vasopressor drugs would interfere in the therapeutic effect of electrochemical therapy.

#### Electrochemical therapy combined with pingyangmycin injection

The surgeon chose general or local anesthesia according to the patient’s systemic condition and needs. We selected unipolar or bipolar ECT according to the size of the lesions (for the lesions ≥5 cm in diameter, select unipolar ECT). For the patients with general anesthesia, we routinely disinfected the surgical site, made the sterile towel spreading and then pulled out the tongue from the mouth, which could expose the malformed lesions; For the patients with local anesthesia, after routine disinfection and sterile towel spreading, the tongue body was pulled out and local anesthetic drugs (lidocaine injection without the vasopressor drugs) were injected into the base and surrounding area of the lesions. Then the operation was performed after the anesthetic took effect. Our team have used the color Doppler ultrasound, enhanced CT or MRI to accurately locate the insertion position of the electrodes in the venous malformations of the tongue before the operation. The cannulas with the platinum electrode needles were inserted from the normal tissue into the base of the venous malformation in order to block the blood flow in the lesions. The surgeon distributed the electrode needles in parallel with the positive and negative electrodes and at intervals (4). The needle distance was 1.0 ~ 1.5 cm, and the inserting depth was adjusted appropriately. When the internal blood return could be seen in the cannula, the needle core was extracted and the platinum electrode needle was inserted. The surgeon must pay attention to appropriate placement of the cannula outside the normal mucosal tissue to avoid the exposure of the electrode needle to burn the normal mucosal tissue during the operation. During the treatment process, we used bipolar ECT to intermittently administer electrochemical therapy and electrical power was applied once every 3 s, which last 15 s each time for each power-on with cumulative electrical power supply for 2–3 min; the treatment dose during the unipolar ECT surgery was 5–10 C/cm^2^. The surgeon could also observe the changes in the lesions, and stop the electrical power when the lesions became hard or the mucosal surface color was normal. The blood and gas accumulation in the lesion cavity was removed by squeezing the lesion. The platinum needles and the cannulas were removed and the needle holes were closed with absorbable suture. Subsequently, the concentration and dose of pingyangmycin were selected according to the patient’s age and the lesion size. The injection method of pingyangmycin was as follows: after routine local disinfection, a syringe for 5 mL was used to inject from the normal mucosal tissue to the tongue venous malformation. Blood in the lesion cavity was drained as far as possible, and the needle head was kept in the lesion. The syringe containing pingyangmycin solution was then replaced and the pingyangmycin solution was evenly injected into the lesion cavity. According to the location of the lesion, the patient’s age, and the extent and depth of the lesion involvement, 3–5 mL pingyangmycin solution was injected. After the injection, the local pinhole was pressed with a sterilized gauze for 10 min to prevent the injection solution from spilling out. If the lesion involved a large area or had a huge volume, a multi-point injection or multiple regional injection was given and then finger or ring pressure was used to compress the lesion region for 15–30 min to block the blood flow and prolong the local residence time of pingyangmycin. Postoperative prophylactic antibiotic therapy wasn’t needed since the procedure were conducted under means that granted biosecurity. Glucocorticoids were given to prevent postoperative swelling. The therapeutic interval was 3 months for electrochemical therapy combined with pingyangmycin injection until the lesion was cured or good curative effect was obtained. At each follow-up visit, the lesion volume, surface color, and texture changes were recorded in detail. The treatment continued or discontinued according to the degree of the lesion regression or adverse reactions, respectively.

#### Local injection of pingyangmycin alone

The concentration and dose of pingyangmycin were selected according to the patient’s age and the lesion size. The injection method of pingyangmycin was as follows: after routine local disinfection, a syringe for 5 mL was used to inject from the normal tongue mucosal tissue to the tongue venous malformation. Blood in the lesion cavity was drained as far as possible, and the needle head was kept in the lesion. The syringe containing pingyangmycin solution was then replaced and the pingyangmycin solution was evenly injected into the lesion cavity. According to the location of the lesion, the patient’s age, and the extent and depth of the lesion involvement, 3–5 mL pingyangmycin solution was injected. After the injection, the local pinhole was pressed with a sterilized gauze for 10 min to prevent the injection solution from spilling out. Small superficial venous malformations could be injected until the surface of the lesion became pale. If the lesion involved a large area or had a huge volume, a multi-point injection or multiple regional injection was given and then finger or ring pressure was used to compress the lesion region for 15–30 min to block the blood flow and prolong the local residence time of pingyangmycin. In our study, small lesions were cured by only one injection, larger lesions were injected between three and five times, and the cumulative injection dose totaled less than 40 mg. The vital signs of the patients were closely observed for at least 30 min after the injection, and those patients without abnormal allergic symptoms and signs were allowed to leave the hospital. Postoperative prophylactic antibiotic therapy wasn’t needed since the procedure were conducted under means that granted biosecurity. Glucocorticoids were given to prevent postoperative swelling. The injection was repeated 15–30 days later according to the regression of the lesion. At each follow-up visit, the lesion volume, surface color, and texture changes were recorded in detail. The treatment continued or discontinued according to the degree of the lesion regression or adverse reactions, respectively.

#### Evaluation criteria of curative effect

We used hemisphere measurement (5) to record the sizes of the lesions before and after the treatment. We used the classification of Achauer et al. as the evaluation criteria of curative effect (6), and the patients’ feedback to evaluate the curative effect. The classification of Achauer et al. was as follows: I(poor) = lesion volume decreased by less than 25%, the size of the lesions did not change or continue to expand; II (moderate) = lesion volume decreased between 26 and 50%; III (good) = lesion volume decreased between 51 and 75%, the appearance markedly improved; and IV (excellent) = lesion volume decreased between 76 and 100%, the lesions disappeared completely and no recurrence occurred after 1 year of follow-up. Among them, grade III (good) and IV (excellent) were rated as effective treatment, and grade I(poor) and II (moderate) were rated as ineffective treatment. The classification of Achauer as the evaluation criteria of curative effect was shown in Table 2.

**Table 2:** The classification of Achauer as the evaluation criteria of curative effect.

Achauer classification	Ineffective treatment		Effective treatment	
	I (Poor)	II (Moderate)	III (Good)	IV (Excellent)
Volume decreased	≤25%	26–50%	51–75%	76–100%

### Statistical method

SPSS 22.0 statistical software was used for analysis, and counting data were analyzed by Chi-Square test. *p* < 0.05 was considered statistically significant.

## Results

### Overall efficacy evaluation

**Figure 1:**
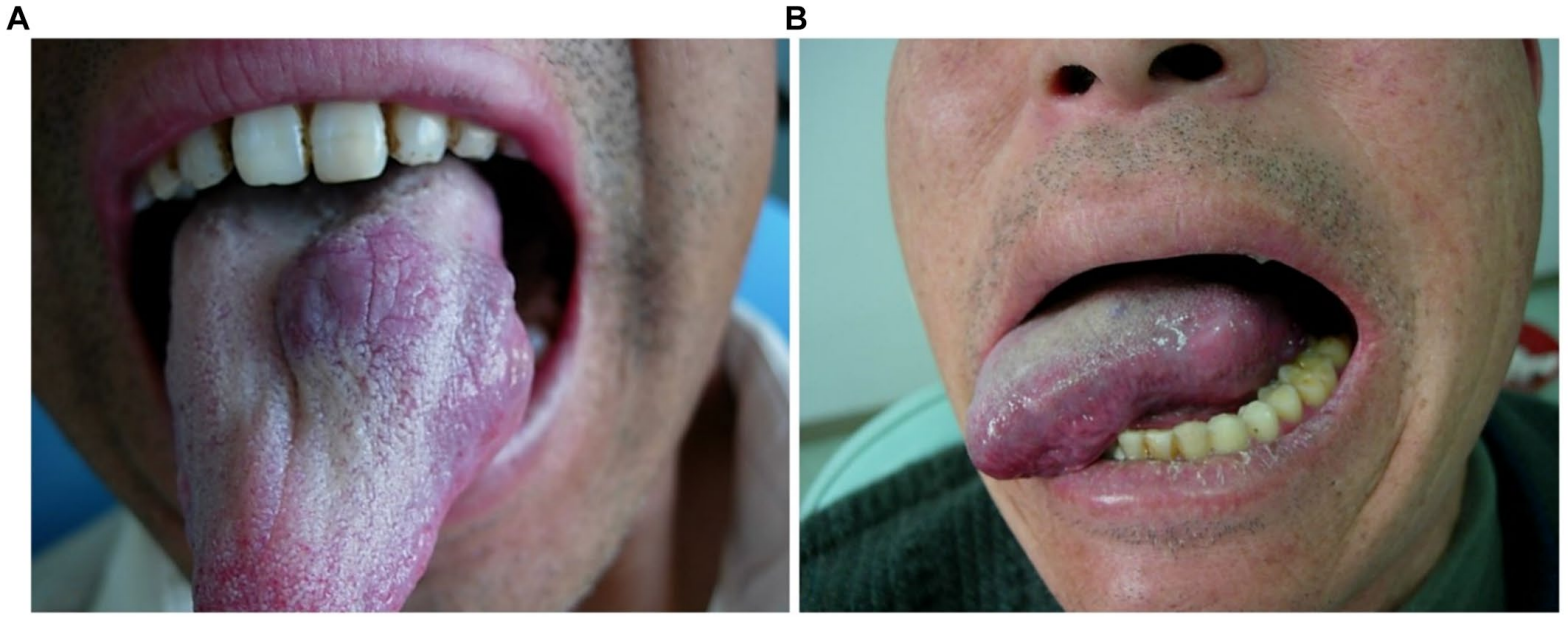
Morphological changes of venous malformation of the tongue in case 1 before and after the treatment. **(A)** Preoperative morphology of venous malformation of the left lingual margin and lingual body. **(B)** Postoperative morphology of the left lingual margin and lingual body 3 months after ECT with PYM injection.

**Figure 2:**
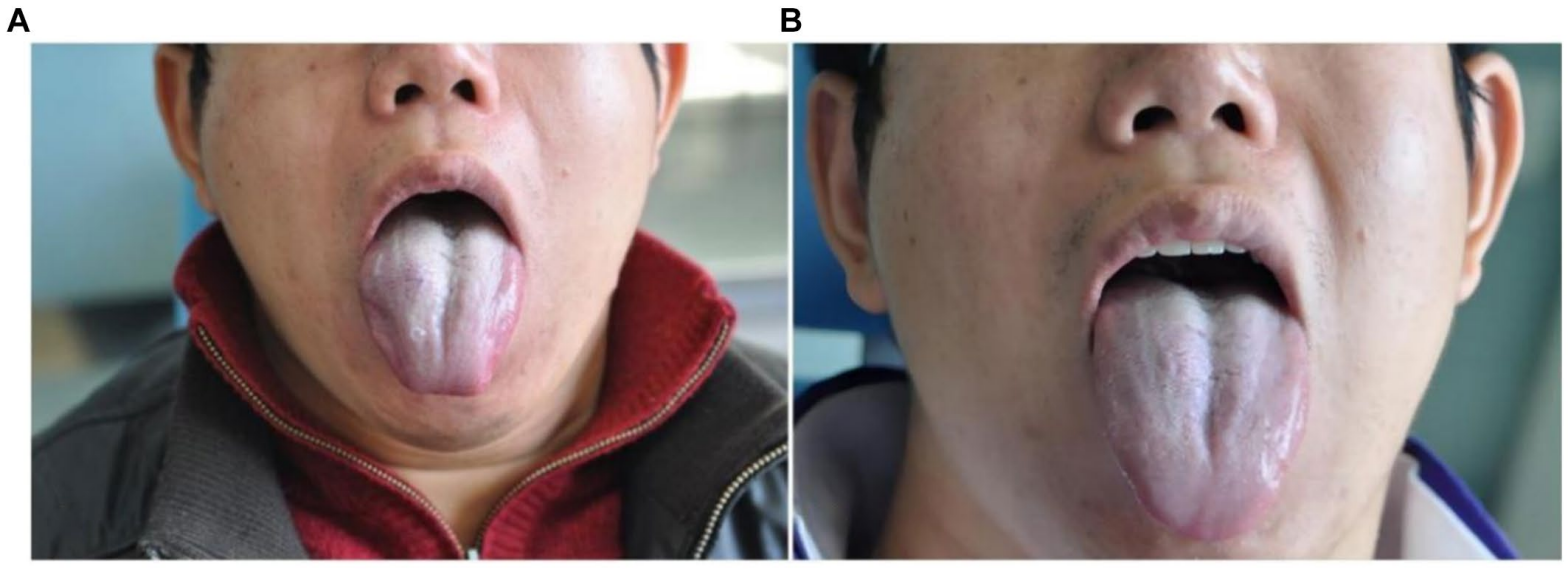
Morphological changes of venous malformation of the right lingual margin and lingual body in case 2 before and after the treatment. **(A)** Preoperative morphology of venous malformation of the right lingual margin and lingual body. **(B)** Postoperative morphology of the right lingual margin and lingual body 3 months after ECT with PYM injection.

**Figure 3:**
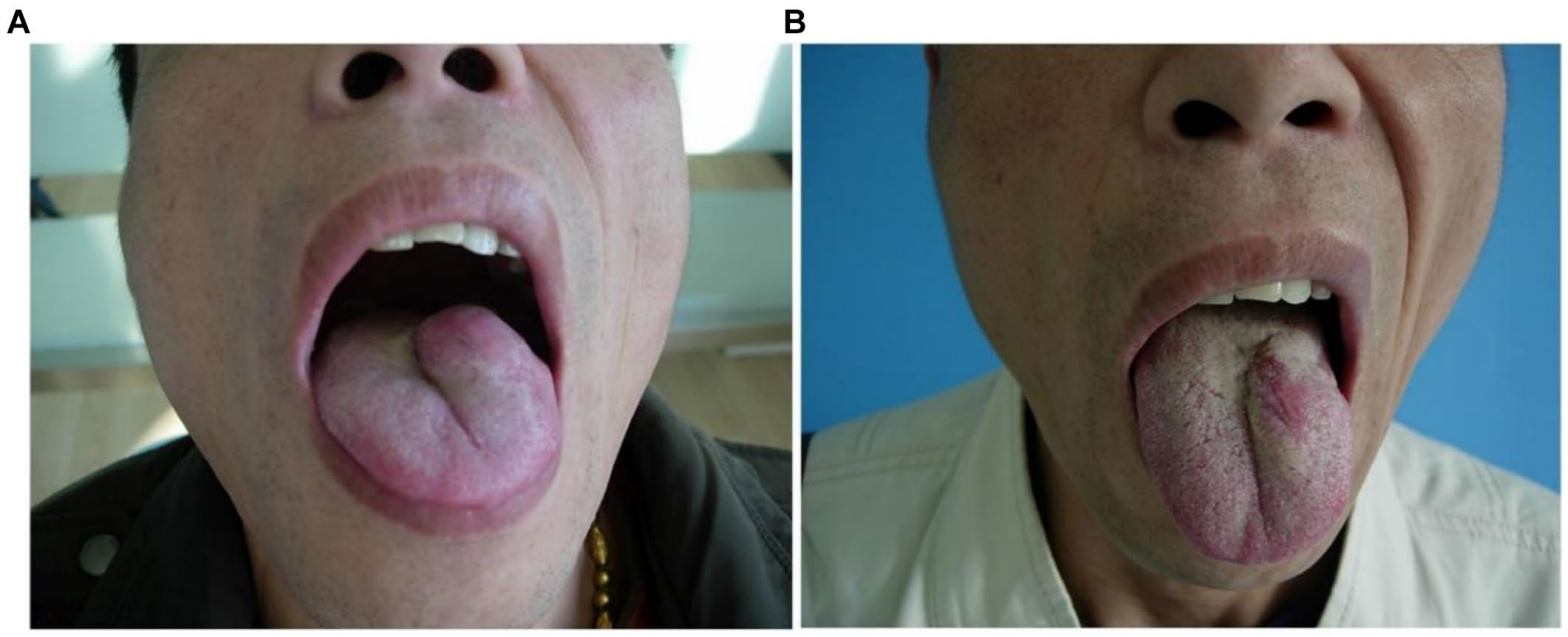
Morphological changes of venous malformation of the left lingual margin and lingual body in case 3 before and after the treatment. **(A)** Preoperative morphology of venous malformation of the left lingual margin and lingual body. **(B)** Postoperative morphology of the left lingual margin and lingual body 3 months after ECT with PYM injection.

**Figure 4:**
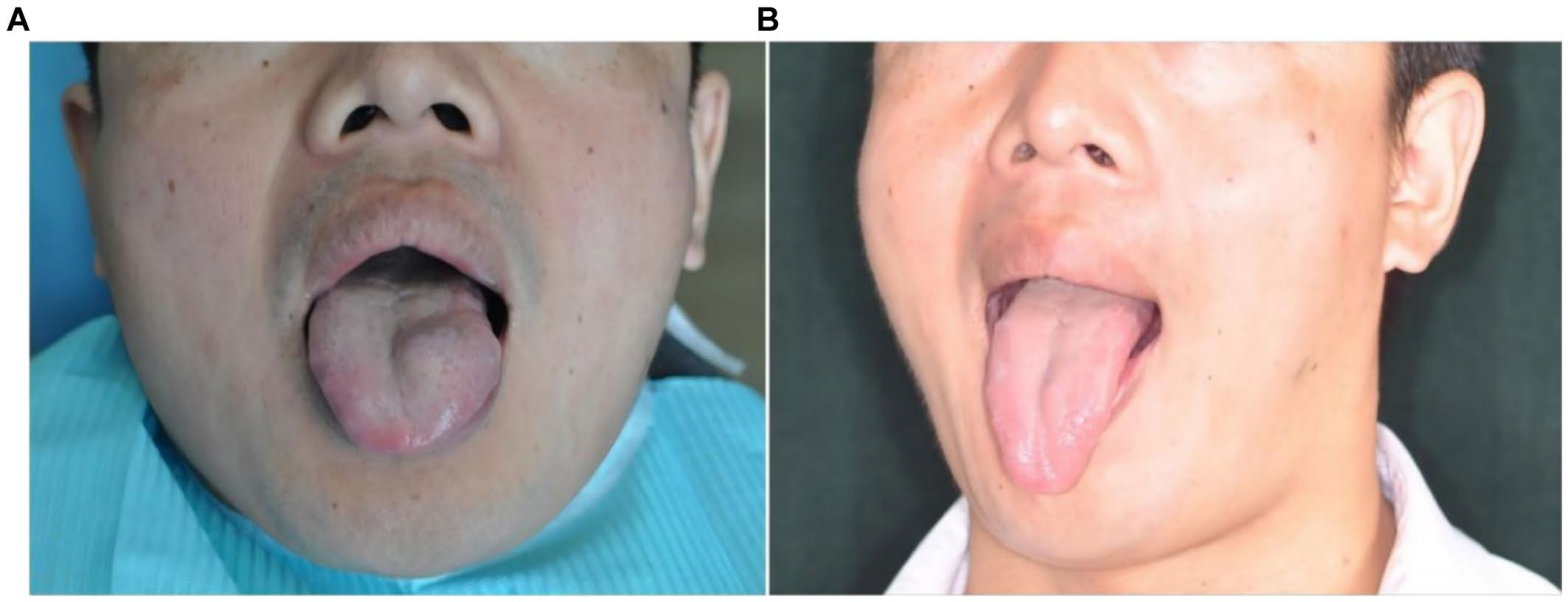
Morphological changes of venous malformation of the left lingual margin and lingual body in case 4 before and after the treatment. **(A)** Preoperative morphology of venous malformation of the left lingual margin and lingual body. **(B)** Postoperative morphology of the left lingual margin and lingual body 3 months after ECT with PYM injection.

**Figure 5:**
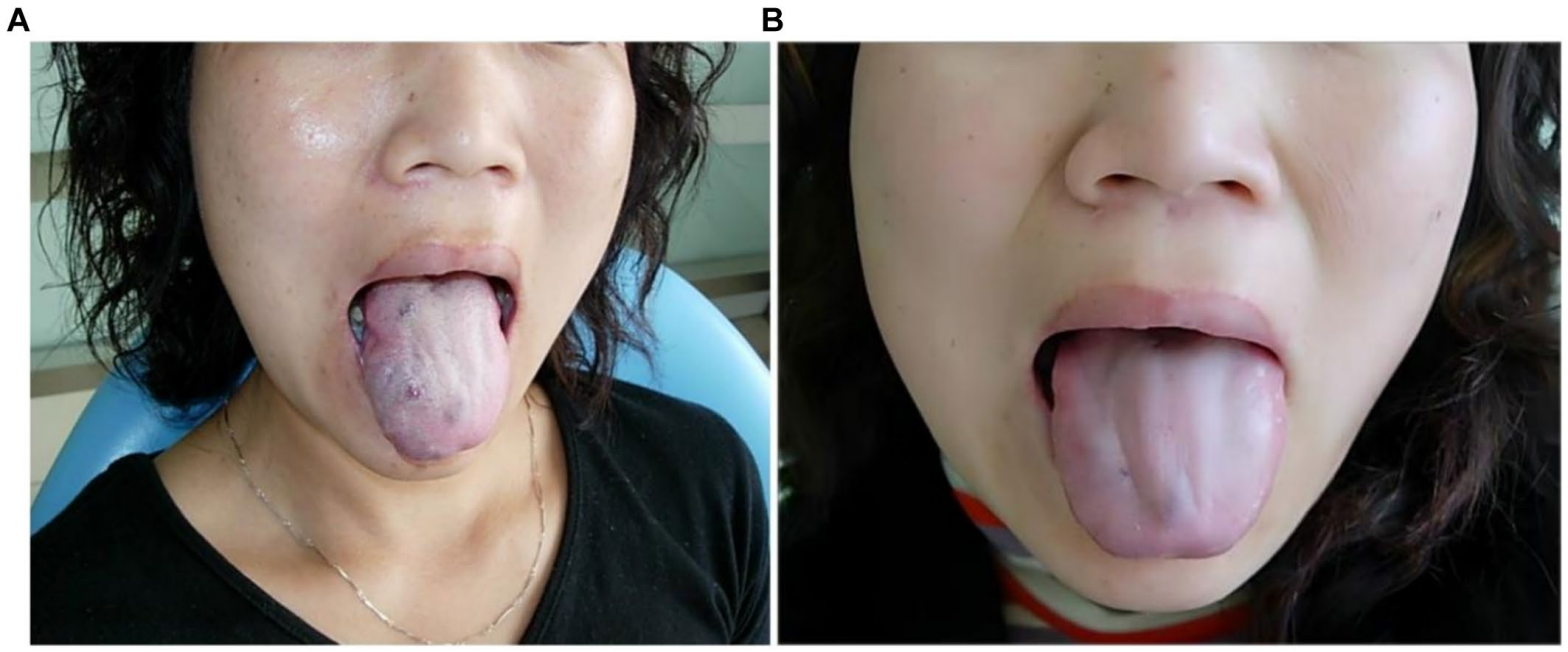
Morphological changes of the multiple venous malformations of the right lingual margin and lingual body in case 5 before and after the treatment. **(A)** Preoperative morphology of the multiple venous malformations of the right lingual margin and lingual body. **(B)** Postoperative morphology of the right lingual margin and lingual body 3 months after ECT with PYM injection.

**Figure 6:**
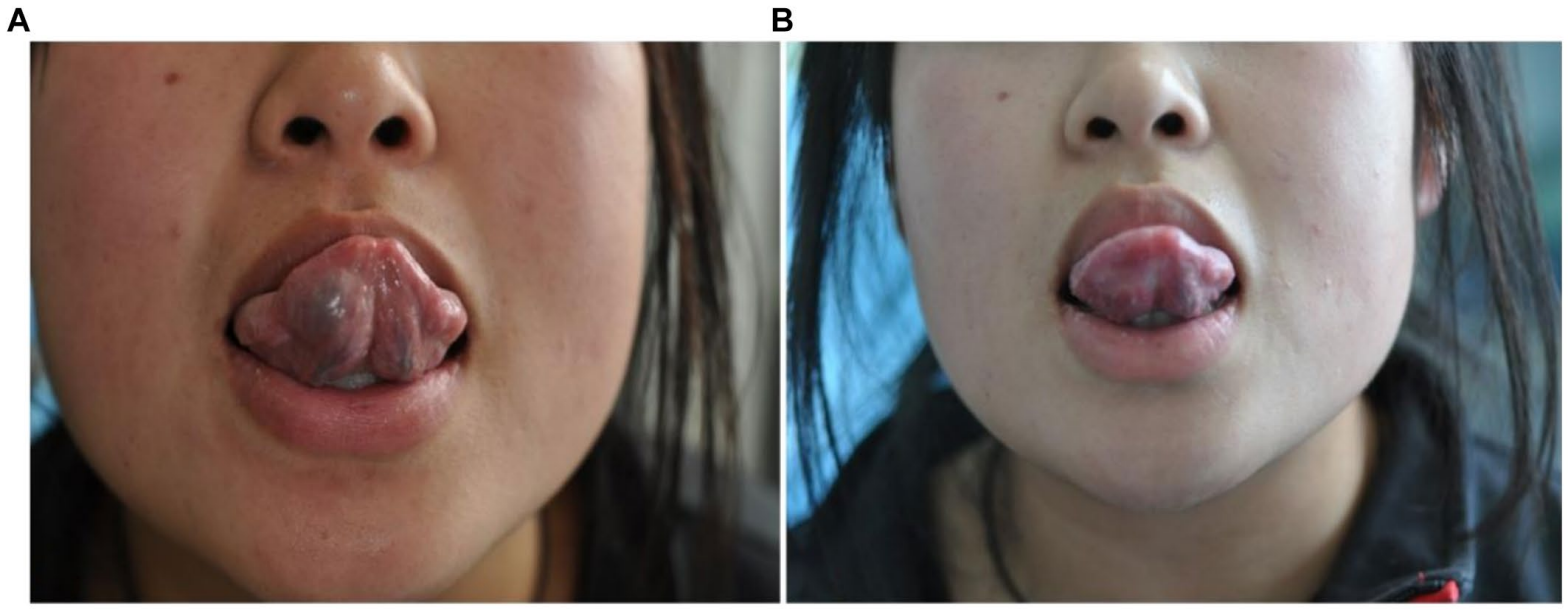
Morphological changes of the venous malformation of the right lingual abdominal in case 6 before and after the treatment. **(A)** Preoperative morphology of the venous malformation of the right lingual abdominal. **(B)** Postoperative morphology of the right lingual abdominal 3 months after ECT with PYM injection.

**Figure 7:**
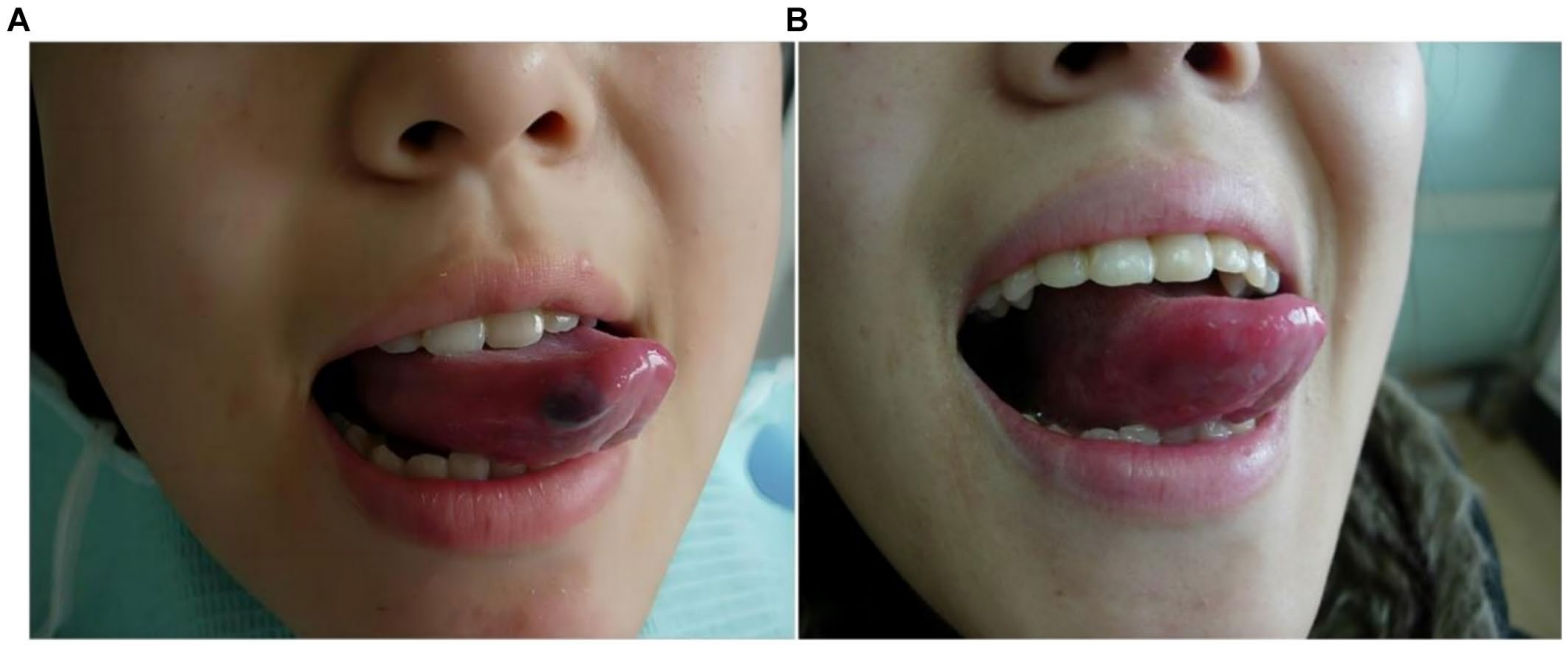
Morphological changes of the venous malformation of the right lingual margin in case 7 before and after the treatment. **(A)** Preoperative morphology of the venous malformation of the right lingual margin. **(B)** Postoperative morphology of the right lingual margin 2 months after local injection of PYM alone.

During the follow-up period from 6 months to 3 years, 60 patients achieved different degree of improvement, with a total effective rate of 85%. 30 patients in the group A received electrochemical therapy (ECT) combined with local injection of pingyangmycin (PYM), with an effective rate of 97%. 30 patients in the group B received local injection of pingyangmycin alone, with an effective rate of 73%. The effectiveness of ECT combined with PYM injection in the group A was significantly higher than that of PYM injection alone in the group B (*p* < 0.05). The treatment results of the two groups with different treatment methods were shown in Tables 3–5. Postoperative symptoms such as local pain, swelling and fever often occurred in the patients, and glucocorticoids were given to prevent postoperative swelling. The postoperative fever (<38°C) was considered to be caused by injection of pingyangmycin, and the symptoms disappeared without special treatment. The symptoms generally disappeared after 5 to 7 days. No mucosal necrosis or nerve damage was found. Postoperative subjective sensation and function of the tongue were normal. The pictures of typical cases were shown in Figures 1–7.

**Table 3:** Comparison of the efficacy of two different methods for the treatment of venous malformations in the tongue [*N* (%)].

Group	*N*	Effective			Ineffective		
		Excellent	Good	Total	Moderate	Poor	Total
ECT + PYM	30	23 (77)	6 (20)	29 (97)	1 (3)	0 (0)	1 (3)
PYM	30	12 (40)	10 (33)	22 (73)	5 (17)	3 (10)	8 (27)
Total	60	35 (58)	16 (27)	51 (85)	6 (10)	3 (5)	9 (15)

ECT + PYM stands for electrochemical therapy combined with local injection of pingyangmycin; PYM stands for local injection of pingyangmycin.

**Table 4:** Summary of the patients’ characteristics and outcomes by electrochemical therapy combined with local injection of pingyangmycin for the treatment of tongue venous malformation.

Patient No.	Gender	Age at Onset, years	Size (cm^a^) prior to treatment	Size (cm^a^) after treatment	Location of the lesion	Type of return flow	Follow up, months	Response
1	M	59	8 × 6 × 5	3.5 × 2 × 1.5	LLM + LLB	High	36	Excellent
2	M	42	5 × 4.5 × 3	0 × 0 × 0.5	RLM + RLB	High	18	Excellent
3	M	56	6 × 5 × 3.5	0 × 0 × 0.5	LLM + LLB	High	24	Excellent
4	M	35	5 × 4.5 × 2.5	0 × 0 × 0	LLM + LLB	High	12	Excellent
5	F	38	6.5 × 6 × 2.5	0 × 0 × 0.5	Multiple	Low	18	Excellent
6	F	26	4.5 × 3.5 × 2	0 × 0 × 0	RLA	High	12	Excellent
7	F	45	4 × 3 × 2.5	2 × 2 × 1	LLM	High	18	Excellent
8	F	15	5.5 × 5 × 2	0 × 0 × 0.5	Multiple	High	12	Excellent
9	F	32	4.5 × 3 × 1.5	0 × 0 × 0	RLA	High	12	Excellent
10	F	52	3.5 × 3 × 3	1 × 1 × 0.5	LLA	Low	12	Excellent
11	F	38	3.5 × 3.5 × 3	0 × 0 × 0	LLB	Low	12	Excellent
12	F	55	7 × 6.5 × 5	0 × 0 × 0	RLM + RLB	Low	36	Excellent
13	M	45	6.5 × 6 × 3.5	0 × 0 × 0.5	Multiple	High	24	Excellent
14	F	48	4 × 3.5 × 2	2.5 × 2 × 1	LLM + LLB	High	18	Excellent
15	F	8	3.5 × 3 × 3	0 × 0 × 0	LLM	Low	6	Excellent
16	F	25	3.5 × 3 × 3	0 × 0 × 0	LLM + LLB	Low	12	Excellent
17	F	38	3.5 × 3 × 3	1.5 × 1.5 × 1	RLM	Low	12	Excellent
18	M	8	4 × 3 × 2	0 × 0 × 0	LLB	Low	12	Excellent
19	M	8	3.5 × 3 × 3	2.5 × 2 × 1	LLM + LLB	Low	12	Excellent
20	F	48	4.5 × 4 × 2.5	1 × 1 × 0.5	RLM + RLB	Low	18	Excellent
21	F	58	4 × 3 × 2.5	2 × 2 × 0.5	RLA + LLA	Low	12	Excellent
22	M	56	6.5 × 5 × 3	0 × 0 × 0.5	LLM + LLB	Low	24	Excellent
23	F	32	5 × 4.5 × 2	0 × 0 × 0	Multiple	Low	18	Excellent
24	M	30	6 × 4.5 × 3	3.5 × 3.5 × 2	RLA	High	24	Good
25	M	35	8 × 6 × 3.5	6 × 4 × 2	RLM + RLB	High	24	Good
26	F	55	6 × 4.5 × 2.5	4.5 × 3.5 × 2	Multiple	Low	12	Good
27	F	68	7 × 6 × 5	5 × 4.5 × 3	LLM + LLB	High	18	Good
28	F	66	8 × 6 × 5	6 × 5 × 3	Multiple	High	24	Good
29	M	33	5.5 × 5 × 2.5	4 × 3.5 × 2	RLM + RLB	High	12	Good
30	F	60	7 × 4.5 × 2.5	5.5 × 4 × 2	Multiple	Low	12	Moderate

^a^Data presented a length/width/depth. M, male; F, female. LLM stands for the left lingual margin; LLB stands for the left lingual body; RLM stands for the right lingual margin; RLB stands for the right lingual body; LLA stands for the left lingual abdominal; RLA stands for the right lingual abdominal; Multiple stands for that the lesions occurred in the multiple locations in the tongue.

**Table 5:** Summary of the patients’ characteristics and outcomes by local injection of pingyangmycin alone for the treatment of tongue venous malformation.

Patient No.	Gender	Age at Onset, years	Size (cm^a^) prior to treatment	Size (cm^a^) after treatment	Location of the lesion	Type of return flow	Follow up, months	Response
1	F	68	8 × 6 × 5	5 × 3 × 2	LLM + LLB	Low	18	Excellent
2	F	35	3.5 × 3 × 3	0 × 0 × 0	RLA	Low	12	Excellent
3	F	46	5 × 4.5 × 3	3 × 3 × 1	RLM + RLB	Low	18	Excellent
4	F	50	6 × 4.5 × 2	2 × 1 × 0.5	LLM + LLB	Low	12	Excellent
5	F	18	3.5 × 3 × 3	0 × 0 × 0	RLA	Low	12	Excellent
6	F	45	3.5 × 3 × 3	0 × 0 × 0	LLM	Low	12	Excellent
7	F	56	5 × 4.5 × 2	0 × 0 × 0	LLM + LLA	Low	24	Excellent
8	F	52	3.5 × 3 × 3	0 × 0 × 0	RLM + RLA	Low	18	Excellent
9	M	8	3.5 × 3.5 × 3	0 × 0 × 0	LLM + LLB	Low	12	Excellent
10	F	36	3.5 × 3 × 3	0 × 0 × 0	LLM	Low	12	Excellent
11	F	40	5 × 3 × 2.5	0 × 0 × 0.5	RLA + LLA	Low	18	Excellent
12	F	20	4.5 × 3 × 2	0 × 0 × 0	RLB	Low	12	Excellent
13	F	55	6 × 4 × 1.5	5 × 3 × 1	Multiple	Low	18	Good
14	F	46	5 × 2.5 × 2	4 × 2 × 1.5	RLM + RLA	Low	24	Good
15	F	48	5 × 3.5 × 2	3 × 3 × 1.5	RLM + RLB	Low	18	Good
16	F	38	5.5 × 4.5 × 2	4 × 3 × 1.5	Multiple	Low	24	Good
17	M	23	3.5 × 3.5 × 2	3 × 2.5 × 1.5	RLA	Low	18	Good
18	M	25	3.5 × 3 × 2.5	3 × 2 × 2	LLM + LLB	Low	12	Good
19	M	12	4.5 × 4 × 2	3.5 × 3 × 1.5	RLA + LLA	Low	12	Good
20	F	35	6 × 3.5 × 2	4 × 2.5 × 2	LLM + LLA	Low	18	Good
21	M	27	5.5 × 5 × 2.5	4.5 × 3.5 × 2	LLM + LLB	Low	12	Good
22	M	32	3.5 × 3 × 2.5	3 × 2.5 × 1.5	RLM + RLB	Low	12	Good
23	F	30	8 × 5.5 × 4.5	7 × 5 × 4	Multiple	High	18	Moderate
24	M	28	8 × 6 × 3	7 × 5.5 × 2.5	RLA + LLA	High	24	Moderate
25	F	55	6 × 5.5 × 3.5	5 × 4.5 × 3	LLM + LLB	High	18	Moderate
26	F	56	3.5 × 3 × 2	3.2 × 2.3 × 2	LLM	High	12	Moderate
27	F	26	5 × 3.5 × 2.5	4 × 3 × 2	LLA	High	12	Moderate
28	M	50	6 × 5 × 3	5 × 4.5 × 3	LLM	High	18	Poor
29	F	48	5.5 × 5 × 3	5 × 5 × 2.5	LLM + LLB	High	12	Poor
30	M	65	8 × 6 × 5	7 × 5 × 3	Multiple	High	12	Poor

^a^Data presented a length/width/depth. M, male; F, female. LLM stands for the left lingual margin; LLB stands for the left lingual body; RLM stands for the right lingual margin; RLB stands for the right lingual body; RLA stands for the right lingual abdominal; LLA stands for the left lingual abdominal; Multiple stands for that the lesions occurred in the multiple locations in the tongue.

### Typical cases

*Case 1*: The patient was a 59-year-old male. The venous malformation of the left lingual margin and lingual body lasted for more than 10 years, and the progressive enlargement of the lesion affected eating and speech. The lesion of the left lingual margin and lingual body recovered after 5 s compression, which was a high-return flow venous malformation. After ECT combined with PYM injection, the lesion was significantly reduced 3 months after the treatment, and completely cured. The pictures of the lesion before and after the treatment were shown in Figure 1.

*Case 2*: The patient was a 42-year-old male. The venous malformation of the right lingual margin and lingual body lasted for more than 6 years, and the progressive enlargement affected eating and speech. The lesion of the right lingual margin and lingual body recovered after 5 s compression, which was a high-return flow venous malformation. After ECT combined with PYM injection, the lesion was significantly reduced 3 months after the treatment, and completely cured. The pictures of the lesion before and after the treatment were shown in Figure 2.

*Case 3*: The patient was a 56-year-old male. The venous malformation of the left lingual margin and lingual body lasted for more than 5 years, and the progressive enlargement of the lesion affected eating and speech. The lesion of the left lingual margin and lingual body recovered after 5 s compression, which was a high-return flow venous malformation. After ECT combined with PYM injection, the lesion was significantly reduced 3 months after the treatment, and completely cured. The pictures of the lesion before and after the treatment were shown in Figure 3.

*Case 4*: The patient was a 35-year-old male. The venous malformation of the left lingual margin and lingual body lasted for more than 3 years, and the progressive enlargement of the lesion affected eating and speech. The lesion of the left lingual margin and lingual body recovered after 5 s compression, which was a high-return flow venous malformation. After ECT combined with PYM injection, the lesion was significantly reduced 3 months after the treatment, and completely cured. The pictures of the lesion before and after the treatment were shown in Figure 4.

*Case 5*: The patient was a 38-year-old female. The multiple venous malformations of the right lingual margin and lingual body lasted for more than 3 years, and the progressive enlargement of the lesions affected eating and speech. The multiple venous malformations of the right lingual margin and lingual body might occasionally cause bleeding. The multiple lesions of the right lingual margin and lingual body did not recover after 5 s compression, which were the low-return flow venous malformations. After ECT combined with PYM injection, the lesions were significantly reduced 3 months after the treatment, and completely cured. The pictures of the lesions before and after the treatment were shown in Figure 5.

*Case 6*: The patient was a 26-year-old female. The venous malformation of the right lingual abdominal lasted for more than 5 years, and the progressive enlargement of the lesion affected eating and speech. The lesion of the right lingual abdominal recovered after 5 s compression, which was a high-return flow venous malformation. After ECT combined with PYM injection, the lesion was significantly reduced 3 months after the treatment, and completely cured. The pictures of the lesion before and after the treatment were shown in Figure 6.

*Case 7*: The patient was a 23-year-old female. The venous malformation of the right lingual margin lasted for more than 2 years, and the progressive enlargement of the lesion affected eating and speech. The lesion of the right lingual margin did not recover after 5 s compression, which was a low-return flow venous malformation. After local injection of pingyangmycin alone, the lesion was significantly reduced 2 months after the treatment, and completely cured. The pictures of the lesion before and after the treatment were shown in Figure 7.

## Discussion

Based on the cell biological characteristics of vascular diseases, Mulliken and Glowacki proposed in 1982 to clearly divide them into two categories: hemangioma and vascular malformation, which has been widely accepted by the scholars around the world. Hemangioma and vascular malformation were two kinds of lesions with different biological characteristics, and there were some essential differences between them in pathology: (1) Hemangioma had the characteristics of the proliferation of vascular endothelial cell. It was a kind of true tumor, and it grew rapidly after birth. The main pathological change of proliferative hemangioma was the proliferation of endothelial cell, which resulted in narrowed vascular lumen. And the endothelial cells on the surface of the lumen were wrinkled and not smooth. (2) Vascular malformations were congenital developmental abnormalities that consist of enlarged dysplastic blood vessels. Vascular malformations did not have the characteristics of the proliferation of vascular endothelial cell. The vascular endothelium and lining of vascular malformations did not have the proliferative tendency. And they did not belong to true tumors. Vascular malformations had the characteristics of thick lumen diameter, smooth lumen wall and rapid blood flow (7). Venous malformation was the most common vascular malformation in oral and maxillofacial region (8). According to the imaging characteristics of the returning veins, venous malformations could be divided into high-return flow and low-return flow types (9, 10). Venous malformations were the most common type of vascular malformations in the tongue (3). Tongue venous malformations might not be detected at birth and grew slowly with age. When combined with infection or external stimulation, the lesions could appear pain, obvious enlargement, swelling, bleeding and other symptoms. If large tongue venous malformations developed to affect eating, speech and breathing, they should be treated as soon as possible.

In the past, the treatment methods for tongue venous malformations included laser, surgical resection, sclerotherapy and interventional embolization, but the above treatment methods had limited indications and unsatisfactory therapeutic effect. Laser was only suitable for superficial mucosa and small venous malformations, but it had poor therapeutic effect on deep and extensive venous malformations (11, 12). Surgical resection was suitable for localized lesions. Invasive lesions required a wide range of resection, which could lead to loss of tongue function, lingual nerve damage, or massive bleeding (13, 14). Interventional embolization therapy was usually used as a palliative and preoperative treatment, and its complications were more severe, including ischemic necrosis of the tissue, persistent severe pain, loss of vital organ function, and even death from heart and lung failure (15).

Electrochemical therapy was established on the basis of the biological closed electric circuits (BCEC) proposed by the Swedish scholar Nordenstrom (16). The mechanism of electrochemical therapy of vascular malformations included: (1) The direct current electrode was directly inserted into the platinum needle in the diseased cavity, and a certain intensity of bioelectric field was formed in the diseased cavity after being energized, which could result in electrolyte disturbance, acid–base imbalance, and local electrochemical and electrophysiological changes. The negatively charged Clˉ ions released by electrolyte NaCl and H_2_O after electrolysis moved to the positive electrode region, which made the pH of the positive electrode region drop to 1–2 (showing strong acidity). The positively charged Na + electrolysed moved to the needle area of the negative electrode, and bonded with OHˉ ion to form strongly alkaline NaOH, which increased the pH of the negative electrode region to 12–13, presenting a strong alkaline state. Red blood cells and platelets in the vascular tissues were seriously damaged and hemagglutinins were released. Then the lesions showed solid changes. (2) Direct current enhanced the permeability of the cell membrane, which caused the ions to move and diffuse in the electric field and produced oxygen and chlorine. The oxygen and chlorine directly killed the diseased cells; (3) Direct current changed the internal and external environment of the cells, destroyed the activity of cell enzymes, and led to the denaturation of the protein; (4) By the electrolysis, spot osmosis, electrophoresis and other functions, the distribution of intercellular ions concentration were changed. There were tissue dehydration and contraction of large blood vessels and capillaries in the positive electrode region. And there was extensive formation of microvascular thrombosis in the positive electrode region. While in the negative electrode region, there were interstitial edema, a large amount of accumulated fluid, compressed capillaries, and obstructed tissue blood flow. (5) According to the literature report, the volume of endothelial cells was significantly reduced and the vascular lumen was blocked at the beginning of electrochemical therapy. After the electrochemical therapy, the structure of the lesion disappeared completely and the endothelial nucleus was dissolved and destroyed. The lesion became a uniform denatured-necrotic tissue. (6) Other studies had shown that electrochemical therapy could induce cell apoptosis, promote the necrosis of tumor, and inhibit the growth of tumor (17–19).

At present, the most used method for venous malformations is the sclerosing agent pingyangmycin, applied as local injection sclerotherapy (11). Pingyangmycin has a chemical structure similar to bleomycin A5, and because of its safety, convenience and effective characteristics, intralesion injection of pingyangmycin has been widely used in the treatment of oral and maxillofacial-head and neck venous malformations (20). Studies have shown that the main mechanisms of pingyangmycin in the treatment of venous malformations were as follows: pingyangmycin played its therapeutic effect by binding to DNA of vascular endothelial cells, which caused DNA strand breakage of vascular endothelial cells and inhibited the metabolism of vascular endothelial cells. This could result in the atrophy and degeneration of vascular endothelial cells and the specific damage to vascular endothelial cells and the vascular wall. It could also induce the proliferation of vascular smooth muscle cells and endothelial cells and thicken the vascular wall, which could narrow and eventually block the vascular lumen. Some studies have also found that pingyangmycin can destroy the vascular endothelial cells and promote the expression of adhesion molecules on the surface of endothelial cells, or release a variety of growth factors. Pingyangmycin can also promote inflammatory cell adhesion, infiltration, and inflammatory chemotaxis, and lead to the release of inflammatory factors and tissue fibrosis factors. This can promote the fibrosis of the tissue and eventually lead to the occlusion of vascular lumen (21, 22).

However, pingyangmycin was a kind of mild sclerosing agent. Due to its short retention time and limited action time in the lesion cavity, local injection of pingyangmycin alone was generally ineffective in the treatment of venous malformations with the high-return flow lesions, and other auxiliary measures or even multiple injections were needed for the deep and extensive venous malformations. In our study, we found that local injection of pingyangmycin alone had excellent therapeutic effect for the superficial mucosal or the localized venous malformations and local injection of pingyangmycin alone could also have very good effect for the low-return flow venous malformations. However, local injection of pingyangmycin alone had very poor therapeutic effect for the large, deep and extensive venous malformations or the high-return flow lesions. 8 patients with high-return flow venous malformations in the group B had poor therapeutic effect by the method of local injection of pingyangmycin alone in our study. The results of our study showed that the effective rate of local injection of pingyangmycin alone was 73%, while that of electrochemical therapy combined with local injection of pingyangmycin was 97%, and the difference between the two groups was statistically significant (*p* < 0.05). 15 patients with high-return flow venous malformations in the group A had very good therapeutic effect by the method of electrochemical therapy combined with local injection of pingyangmycin. On the one hand, electrochemical therapy led to the swelling, degeneration and destruction of vascular endothelial cells in the lesion. It could also increase the permeability of cell membrane and make widespread thrombosis in the lesion. On the other hand, pingyangmycin stayed in the lesion cavity for a long retention time along with electrochemical therapy. The concentration of pingyangmycin in the lesion cavity increased and accelerated the destruction of vascular endothelial cells. While pingyangmycin was playing its therapeutic effect in the lesion, pingyangmycin also aggravated the chemical killing effect of direct current of electrochemical therapy, destroyed the pathological structure more thoroughly, promoted the coagulation in the lesion cavity, and led to the complete disappearance of blood flow. Finally the sclerosed vascular tissue was absorbed, so as to achieve the excellent therapeutic effect.

The common adverse reactions of pingyangmycin injection in the treatment of venous malformations are usually mild, and include swelling and pain, fever, skin reaction (local itching), gastrointestinal reaction, and local ulceration and necrosis, while anaphylactic shock, pulmonary fibrosis, and leucopenia are rare (23, 24). To reduce the occurrence of adverse reactions, we added lidocaine to the solution to reduce swelling and pain, and meanwhile lidocaine could also reduce the occurrence of anaphylactic reaction. Postoperative glucocorticoid therapy could reduce the swelling response. In our treatment, the swelling response usually went away in about 1 week. When the temperature of the patients with fever was higher than 38.5°C, the symptoms were improved after taking antipyretic drugs. The biggest concern of pingyangmycin injection treatment for a long time was its pulmonary toxicity, which was closely related to the injection dose (24). Generally, the total dose used for the treatment of vascular malformations was not more than 160 mg, and the dosage of pingyangmycin in our study was far less than 160 mg, so there was no toxicity to the lung. The patients did not develop pulmonary fibrosis in our study. The adverse reactions of electrochemical therapy in the treatment of vascular malformations were rare, and some adverse reactions might occur clinically which included postoperative swelling, fever (<38°C), bleeding, infection, and nerve damage at the lesion site. The above adverse reactions could be restored to normal after symptomatic treatment.

Based on our treatment experience for venous malformation of tongue by electrochemical therapy combined with local injection of pingyangmycin, the following conclusions were drawn: (1) Electrochemical therapy of tongue venous malformation needed to be performed by the trained professional surgeons to ensure the safety of the patients. It was important to have a complete history and thorough examination before performing any surgery. (2) According to our experience, for the lesions in the posterior 1/3 of the tongue, the use of electrochemical therapy under general anesthesia was better and safer. The lesions could be fully exposed, and the treatment was more accurate. (3) The needles in parallel with the positive and negative electrode were inserted evenly at intervals, and the distance between the electric needles was 1.5 cm. (4) The relationship between the lesion and the peripheral nerve should be clearly determined, and the peripheral nerve should be avoided when inserting the electric needles. (5) The surgeon should properly place the insulated cannulas with the platinum electrode needles, slowly increase the current and voltage during the treatment and reasonably set the total amount of electricity required for the treatment to avoid the skin burns. (6) In the course of the treatment, sterile gauze were used to evenly squeeze the diseased tissue. The surgeon tried to drain the blood and gas in the lesion, make the lesion cavity smaller and restore the shape and flatness of normal tissue as far as possible. (7) The treatment time of electrochemical therapy was mainly determined by the size of the lesions. Generally, when the surface skin color of the lesion turned white or the lesion became slightly hard, and when there was brownish black viscous liquid flowing out of the cannulas of the platinum electrode needles, it can be regarded as an indication of electrochemical therapy cessation. (8) The needle holes after the removal of the electric needles should be properly treated, such as gauze compression of the needle holes or non-invasive suture ligation to avoid secondary bleeding. (9) The size of the lesions and the age of the patients determined the concentration and the injection times of pingyangmycin. For tongue venous malformation, the usual injection concentration for adults was 1.6 mg/mL. The injection concentration for children was 1.0 mg/mL. If the lesion involved a large area or had a huge volume, a multi-point injection or multiple regional injection was given. In our study, small lesions were cured by only one injection, and larger lesions were injected between three and five times.

In conclusion, electrochemical therapy combined with local injection of pingyangmycin for the treatment of tongue venous malformation had the advantages of minimally invasive treatment, few postoperative adverse reactions, and significant clinical efficacy, which was easy to be accepted by the patients. This method is safe and effective for all kinds of tongue venous malformations. It is the first choice of treatment especially for the high-return flow venous malformations in the tongue. The treatment modality is worthy of further promotion in clinic. However, this study has several limitations, including the small sample size and the retrospective design. In the future, additional prospective randomized studies with the large sample size are necessary to confirm the efficacy and safety of electrochemical therapy combined with local injection of pingyangmycin for the treatment of tongue venous malformation.

## Data Availability

The original contributions presented in the study are included in the article/supplementary material, further inquiries can be directed to the corresponding author.
